# Supplementary material for: Causal Relationship between Aging and Anorexia Nervosa: A White-Matter-Microstructure-Mediated Mendelian Randomization Analysis
Source: Biomedicines. 2024 Aug 16;12(8):1874. doi: 10.3390/biomedicines12081874 (PMC11351342; doi:10.3390/biomedicines12081874)
Supplement: Supplementary file 1 [file biomedicines-12-01874-s001.zip › Supplementary Figure/Figure S4/Singe SNP plot of MR analysis for TL to ubm-b-1919.pdf]

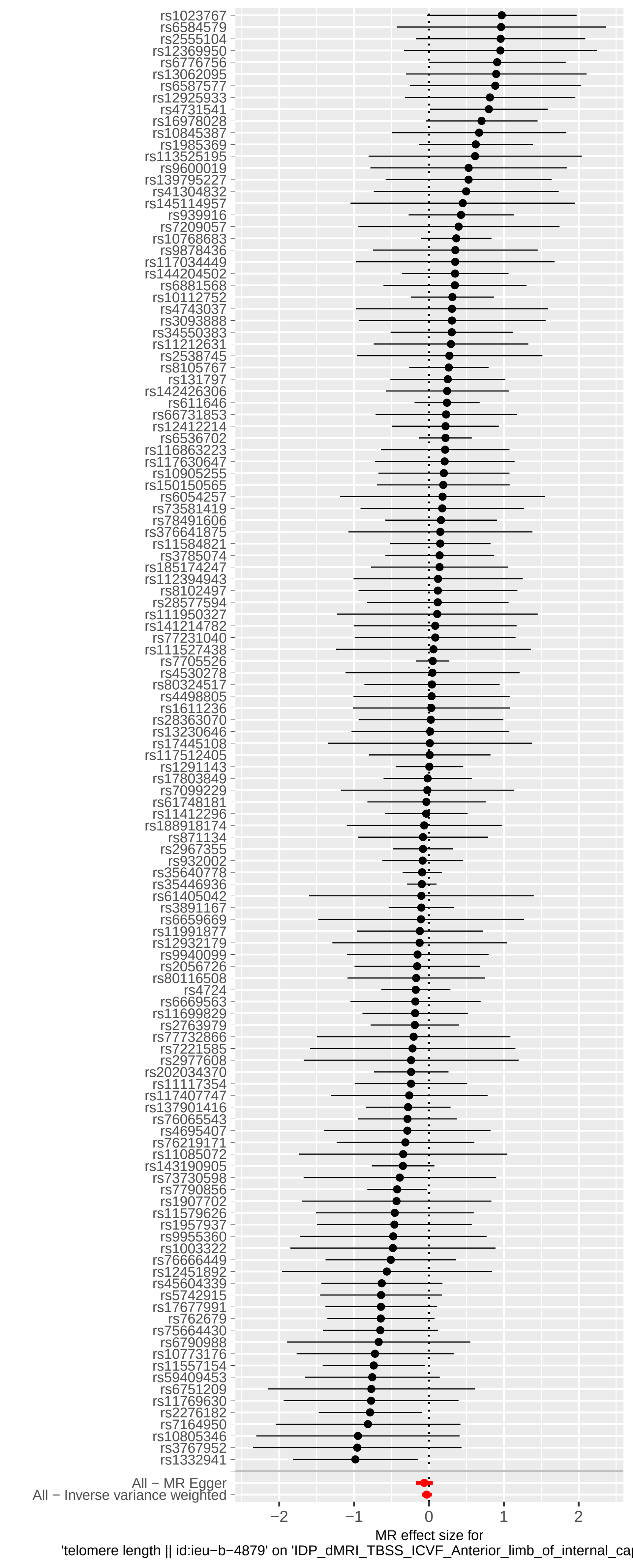
rs1023767
rs6584579
rs2555104
rs12369950
rs6776756
rs13062095
rs6587577
rs12925933
rs4731541
rs16978028
rs10845387
rs1985369
rs113525195
rs9600019
rs139795227
rs41304832
rs145114957
rs939916
rs7209057
rs10768683
rs9878436
rs117034449
rs144204502
rs6881568
rs10112752
rs4743037
rs3093888
rs34550383
rs11212631
rs2538745
rs8105767
rs131797
rs142426306
rs611646
rs66731853
rs12412214
rs6536702
rs116863223
rs117630647
rs10905255
rs150150565
rs6054257
rs73581419
rs78491606
rs376641875
rs11584821
rs3785074
rs185174247
rs112394943
rs8102497
rs28577594
rs111950327
rs141214782
rs77231040
rs111527438
rs7705526
rs4530278
rs80324517
rs4498805
rs1611236
rs28363070
rs13230646
rs17445108
rs117512405
rs1291143
rs17803849
rs7099229
rs61748181
rs11412296
rs188918174
rs871134
rs2967355
rs932002
rs35640778
rs35446936
rs61405042
rs3891167
rs6659669
rs11991877
rs12932179
rs9940099
rs2056726
rs80116508
rs4724
rs6669563
rs11699829
rs2763979
rs77732866
rs7221585
rs2977608
rs202034370
rs11117354
rs117407747
rs137901416
rs76065543
rs4695407
rs76219171
rs11085072
rs143190905
rs73730598
rs7790856
rs1907702
rs11579626
rs1957937
rs9955360
rs1003322
rs76666449
rs12451892
rs45604339
rs5742915
rs17677991
rs762679
rs75664430
rs6790988
rs10773176
rs11557154
rs59409453
rs6751209
rs11769630
rs2276182
rs7164950
rs10805346
rs3767952
rs1332941
All − MR Egger
All − Inverse variance weighted
−2
−1
0
1
2
MR effect size for
'telomere length || id:ieu−b−4879' on 'IDP_dMRI_TBSS_ICVF_Anterior_limb_of_internal_ca
